# Regenerative Strategies for Craniofacial Disorders

**DOI:** 10.3389/fphys.2012.00453

**Published:** 2012-12-14

**Authors:** Catharine B. Garland, Jason H. Pomerantz

**Affiliations:** ^1^Department of Surgery, Division of Plastic and Reconstructive Surgery, University of California San FranciscoSan Francisco, CA, USA; ^2^Department of Orofacial Sciences, University of California San FranciscoSan Francisco, CA, USA; ^3^Craniofacial and Mesenchymal Biology Program, University of California San FranciscoSan Francisco, CA, USA; ^4^Eli and Edythe Broad Center of Regeneration Medicine and Stem Cell Research, University of California San FranciscoSan Francisco, CA, USA

**Keywords:** regeneration, craniofacial, stem cell, satellite cell, fat transfer, facial nerve

## Abstract

Craniofacial disorders present markedly complicated problems in reconstruction because of the complex interactions of the multiple, simultaneously affected tissues. Regenerative medicine holds promise for new strategies to improve treatment of these disorders. This review addresses current areas of unmet need in craniofacial reconstruction and emphasizes how craniofacial tissues differ from their analogs elsewhere in the body. We present a problem-based approach to illustrate current treatment strategies for various craniofacial disorders, to highlight areas of need, and to suggest regenerative strategies for craniofacial bone, fat, muscle, nerve, and skin. For some tissues, current approaches offer excellent reconstructive solutions using autologous tissue or prosthetic materials. Thus, new “regenerative” approaches would need to offer major advantages in order to be adopted. In other tissues, the unmet need is great, and we suggest the greatest regenerative need is for muscle, skin, and nerve. The advent of composite facial tissue transplantation and the development of regenerative medicine are each likely to add important new paradigms to our treatment of craniofacial disorders.

## Introduction

As the field of regeneration biology progresses, new strategies will develop for treating craniofacial disorders. Craniofacial disorders are unique in that they affect multiple tissues simultaneously and occur across the full spectrum of patient age and development. Treatments for craniofacial disorders have advanced remarkably over the previous century. However, as with all reconstructive approaches, these treatments remain imperfect. The hurdles remaining are generally related to improving our ability to faithfully “replace like with like” and to minimize and eliminate treatment-associated morbidity. The nascent field of regenerative medicine offers promise to achieve some of these goals.

Regeneration is a specific process, different from “healing.” For the purpose of this review, “regeneration” refers to the replacement of human cells or tissues by like cells, reestablishing the original form and function (Mason and Dunnill, 2008). The term regeneration refers to different mechanisms in different tissues. Regeneration may involve the proliferation and differentiation of stem cells within tissue (Greenow and Clarke, 2012; King and Newmark, 2012). For example, this type of regeneration occurs in skin after partial thickness burn injury. Regeneration can also refer to distinct processes, such as axonal regeneration in peripheral nerves, that does not involve direct cell division and proliferation, but are reliant upon supporting cells for regeneration to occur (Zochodne, 2012). In contrast to regeneration, the healing of injuries in humans replaces injured tissue with a collagen-dense scar. In healing, the resultant tissue differs from the native tissue in gross and histologic appearance, strength and stiffness, and function (e.g., scarred muscle has diminished contractility, and scarred skin has diminished sensation and sweating).

A number of evolutionary hypotheses exist as to why adult mammals heal by scar formation after injury, while certain other vertebrates such as salamanders have a remarkable ability to regenerate solid tissues, including complete limbs, without scar. In mammals, including humans, significant regenerative capacity exists primarily during fetal and perinatal development. One hypothesis is that healing by scar formation conferred an evolutionary survival advantage in mammals. The rapid deposition of fibrotic tissue to seal a wound prevents infection and protects vital structures, but also may actively prevent regeneration (Brockes and Kumar, 2008; Gurtner et al., 2008). In support of this, inhibiting the fibrotic response after spinal cord injury in mice permits axonal regeneration (Stichel et al., 1999; Klapka and Muller, 2006). Another proposed teleological hypothesis is that mammals have evolved more stringent negative regulation of cellular growth control as part of advanced tumor suppressor mechanisms. This could confer a survival advantage at the expense of regenerative capacity (Blau and Pomerantz, 2011). A logical extension of such observations is that regenerative capacity may have been lost in higher organisms in favor of tissue stability, avoidance of infection, cancer prevention, and longevity. However, an alternate hypothesis is that regeneration was not lost as mammals evolved, but rather that regeneration separately evolved in certain species. Some evidence supporting this theory is the discovery of specific proteins involved in regeneration that are unique to salamanders (Garza-Garcia et al., 2010). It is possible that each of these hypotheses is partly true. A better understanding of the regenerative mechanisms of both lower vertebrates and developmentally immature mammals may inform our approaches to mammalian regeneration.

Regenerative medicine has emerged as “the process of creating living, functional tissues to repair or replace tissue or organ function lost due to age, disease, damage, or congenital defects” (http://report.nih.gov/NIHfactsheets/ViewFactSheet.aspx?csid=62). Plastic and reconstructive surgery, and the craniofacial subspecialty, is an old field of medicine with an almost identical focus: repairing or reconstructing defects of form and function in diverse tissues and patients (American Board of Plastic Surgery, 2012). The purpose of this review is to discuss where novel approaches to treat craniofacial conditions are most needed. This review will examine how regenerative strategies may improve upon current reconstructive practices. We consider the spectrum of craniofacial disorders and how craniofacial tissues differ from their analogs elsewhere in the body in function and embryologic origin. We then present a problem-based approach to illustrate current strategies for treatment, as well as what we consider the most critical regenerative goals for craniofacial bone, fat, muscle, nerve, and skin. Regenerative strategies for teeth, cartilage, salivary glands, and sensory organs contributing to sight, hearing, smell, and taste, all of importance to craniofacial medicine, are beyond the scope of this review and are addressed in other excellent reviews (For teeth, see: Mao et al., 2006; Huang et al., 2009; Yildirim et al., 2011; Machado et al., 2012; for auricular cartilage, see: Bichara et al., 2012; for salivary glands, see: Kagami et al., 2008; for retina, see: Lamba et al., 2008; Singh and MacLaren, 2011; for inner ear, see: De Felipe et al., 2011; Okano and Kelley, 2012; for olfactory, see: Goldstein and Lane, 2004; Costanzo and Yagi, 2011; for taste, see: Miura and Barlow, 2010). In some cases, our current treatments and innate healing responses provide adequate solutions. In other craniofacial disorders, regenerative medicine may lead to improved tissue appearance and function, and decreased morbidity.

## The Complex Functions of Craniofacial Tissues in Health and Disease

The face has a remarkably complex function in humans. The tissues of the face receive and transmit tremendous amounts of information each day. The cranial nerves receive information from all five senses. Muscles of the face respond to stimuli with complex expressions, and are responsible for rapid movements of the eyes and forceful movements of the jaw in mastication. In addition, bones of the skull protect the brain and orbits. Facial appearance is a fundamental component of individuality. Craniofacial disorders lead to abnormalities in a wide range of patients and tissues that disrupt these functions (Table 1). These problems can be physically, emotionally, and socially disabling. Given the complex nature of craniofacial function and disease, recreating these tissues is a daunting task. Even the most sophisticated of our current approaches do not fully reproduce the fine complex function and form that is the hallmark of craniofacial anatomy and physiology. Newer regenerative approaches may offer paradigm changes toward this goal. In developing regenerative strategies, the tissues must be considered individually, as well as in combination with each other. Some endogenous tissue repair mechanisms may provide solutions for regenerating tissues. In other cases, true regeneration may not be necessary to achieve an excellent outcome.

**Table 1:** **Examples of craniofacial disorders and corresponding unmet “regenerative” needs**.

Disease	Tissue defects	Current strategies	Regenerative need
**CONGENITAL**			
Craniosynostosis	Early bony suture fusion, aberrant skull growth if untreated	Successful bone regeneration after surgery if treated before age one	Promoting complete regeneration of the skull after surgery in all cases
Cleft lip/palate	Deficiency of palatal fusion including bone, muscle, and mucosa	Staged surgical repairs	Mucosa, without scarring that limits bone growth and causes maxillary deficiency
	Secondary deformities from inadequate growth after surgical intervention	Alveolar bone grafting	Elimination of bone graft donor site morbidity
Craniofacial microsomia	Deficient bone and soft tissue development of the face	Distraction osteogenesis Fat grafting Free tissue transfer	Multiple structures are hypoplastic: bone, muscle, skin, cartilage, nerve Achieving normal appearance
Microtia	Deficient and abnormal ear cartilage formation	Reconstruction with rib graft or alloplastic material	A functional reproduction of a normal ear without requiring a rib graft, and with less scarring
Moebius	Bilateral facial paralysis due to underdevelopment of cranial nerves	Free tissue transfer	Cranial nerve generation, or regeneration Development of target muscles
**TRAUMATIC**			
Burn	Need for full skin coverage	Split-thickness skin grafting	Regenerated complete skin organ (epidermis, dermis, and appendages)
	Secondary deformities associated with scar contracture and loss of cartilaginous support	Fat and skin grafting to contractures	Supple, well-vascularized skin replacement with underlying cartilage framework
Fractures	Bone gaps occasionally present due to trauma, malunion, or non-union	Fixation Bone grafts	Regeneration of large defects
Soft tissue atrophy or tissue loss due to injury	May affect fat, muscle, skin, cartilages, mucosa, or nerves	Fat grafting Free tissue transfer	“Composite tissue” regeneration to replace subtle and complex form and function
		Skin grafting	
		Face transplantation	
**ONCOLOGIC**			
Oropharyngeal or other facial cancers	Bone, soft tissue, muscle, and nerve may be radically resected	Free tissue transfer	“Composite tissue” regeneration to replace subtle and complex form and function
Radiation	Negatively affects skin and soft tissue elasticity and healing; causes osteoradionecrosis	Fat grafting Bone grafting	Skin regeneration Bone regeneration
**IDIOPATHIC**			
Bell’s palsy	Facial nerve paralysis Secondary muscle denervation and atrophy	Micro-neurovascular free muscle transfer	Nerve and muscle regeneration to achieve complex function of multiple muscles
Parry-Romberg/progressive hemifacial atrophy	Progressive loss of soft tissue, nerve, muscle	Fat grafting	Fat regeneration Nerve and muscle regeneration
**AGING**			
	Fat atrophy	Fat grafting	Rejuvenation of skin quality
	Loss of skin elasticity	Skin resurfacing	Rejuvenation of fat quantity and location
	Changes in skin pigmentation	

## The Unique Embryologic Origins of Craniofacial Tissues and the Role of Neural Crest Cells

**Figure 1:**
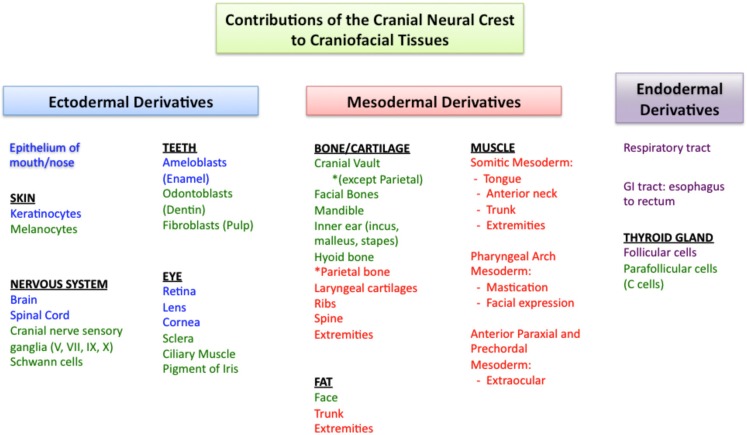
**Cranial neural crest cells have unique contributions to tissues of the face and head**. Ectodermal derivatives are in blue, mesodermal derivatives in red, and endodermal derivatives in purple. In green are the components of these tissues that develop primarily from cranial neural crest cells. In the face and head, cranial neural crest cells contribute to bone, cartilage, and fat, while this is not the case in the trunk and extremities. While all of the craniofacial muscles arise from the mesoderm (red), note that different muscle groups develop from different regions of the mesoderm. References to the developmental origins of the structures in this figure are located throughout the manuscript.

One reason that craniofacial disorders manifest differently from disorders in the trunk and extremities may relate to the distinct embryologic origins of the craniofacial tissues. They subsequently have distinct gene expression patterns and physiology. Understanding these differences may be important for inducing regeneration of craniofacial tissues. Studies of regeneration across phyla suggest that reactivation of developmental signaling pathways is a common theme (reviewed in Sanchez Alvarado and Tsonis, 2006). Therefore, regeneration might be expected to recapitulate the complex interactions of the ectoderm, mesoderm, and endoderm that form the pharyngeal arches, as well as the generation of critical structures by cranial neural crest cells (Figure 1).

Cranial neural crest cells have unique features and play a critical role in the development of the face and head (Le Lièvre and Le Douarin, 1975; Gitton et al., 2010; Grevellec and Tucker, 2010; Cordero et al., 2011; Le Douarin and Dupin, 2012). The dentin-secreting odontoblasts of teeth are exclusively derived from cranial neural crest cells (Lumsden, 1988). While other populations of neural crest cells also contribute to neurons, ganglia, and pigment cells, only cranial neural crest cells are able to form cartilage and bone (Couly et al., 2002; Le Douarin et al., 2007; reviewed in Hall and Gillis, 2012). Most bones of the body are derived from the mesoderm and ossify by endochondral ossification. In contrast, the bones of the face and much of the cranial vault originate from neural crest cells and undergo intramembranous ossification during development (Couly et al., 1993; Jiang et al., 2002; Levi et al., 2012). Cranial neural crest cells are also the primary contributor to fibro adipogenic progenitor cells in the face, whereas fibro adipogenic progenitor cells are of mesodermal origin in the trunk (Lemos et al., 2012). Fibro adipogenic progenitor cells give rise to adipocytes, contribute to fibrofatty infiltration in tissues, and some reports suggest that they may function in concert with muscle precursor cells to facilitate muscle differentiation after injury (Joe et al., 2010).

Since neural crest cells exert a major influence on craniofacial development, are they also mediators of healing potential and disease? If this were the case, one implication would be that engineered or transplanted tissue replacements would either need to be derived from neural crest sources, or be able to derive the phenotypes and perform the functions of cranial neural crest derivatives. The following examples illustrate these considerations. In the skull, it is possible that the unique origin and ossification of craniofacial bones from neural crest cells may be optimized for the massive skull growth occurring in infancy (Jiang et al., 2002). It has also been suggested that the origin of different skull bones influences their healing potential. For example, frontal bone derived from neural crest cells regenerated to fill a defect more rapidly than parietal bone derived from paraxial mesoderm in both juvenile and adult mice (Quarto et al., 2010). In contrast to the differences seen in bone, fibro adipogenic progenitor cells from both the face and trunk appear to exhibit a similar phenotype, differentiation potential, and response to muscle damage despite differences in gene expression (Lemos et al., 2012). Finally, the preference for certain diseases to uniquely affect the face may be attributed to defects in cranial neural crest cell number or function. Neural crest cells have been directly associated with several craniofacial malformations. Treacher Collins syndrome (OMIM 154500), characterized by facial bone hypoplasia, ear deformities, and colobomas of the eyelids, is caused by mutations in *TCOF1* that results in a decrease in the number of neural crest cells (Trainor, 2010). In CHARGE syndrome (OMIM 214800), mutations in *CHD7* are implicated in affecting neural crest cell migration. Dysfunction of neural crest cell migration is also implicated in Waardenburg syndrome, type 2D (OMIM 608890) and Mowat–Wilson syndrome (OMIM 235730; reviewed in Cordero et al., 2011). Neural crest cells may also be involved in fat dystrophies that uniquely affect the face, such as congenital infiltrating lipomatosis (Chen et al., 2002). Other lipodystrophies such as Dunnigan–Kobberling syndrome (OMIM 151660) affect the trunk and extremities, but spare the face. These conditions highlight that a somatic mutation results in distinct phenotypes among craniofacial and body tissues and reinforces the notion that faithful generation or engineering of craniofacial structures may require unique building blocks.

## Different Sets of Craniofacial Muscles Arise from Different Regions of Mesoderm

Like other muscles, craniofacial muscles are also derived from mesoderm, however, groups of craniofacial muscles arise from distinct regions of mesoderm (reviewed in Noden and Francis-West, 2006; Figure 1). Somitic mesoderm forms much of the muscle of the trunk and extremities, but in the face, only the muscles of the tongue and anterior neck are derived from the somites. In contrast, the muscles of mastication and facial expression arise from pharyngeal arch mesoderm, where they develop in close association with the neural crest-derived bones and tendons (Grenier et al., 2009). Finally, extraocular muscles arise from anterior paraxial and prechordal mesoderm (Noden and Francis-West, 2006; Sambasivan et al., 2009). The differences in embryologic origin of face muscles and body muscles are accompanied by differences in the signaling molecules that trigger muscle differentiation in these locations (Sambasivan et al., 2009; reviewed in Kelly, 2010).

Similarly, satellite cells, the tissue-resident muscle stem cells, have different gene expression patterns and characteristics in the face compared with the body. For example, in the trunk, satellite cells express Pax7 and Pax3 (Relaix et al., 2005). However, only Pax7 is expressed in the muscles of the face (Harel et al., 2009; Otto et al., 2009; Kelly, 2010). Satellite cell frequency in muscle fibers also differs. Extraocular, laryngeal, and masseteric muscles have a greater frequency of satellite cells than other skeletal muscles (McLoon et al., 2007). Furthermore, uninjured extraocular and laryngeal muscles contain significant populations of activated satellite cells under normal conditions. These muscles have a high level of basal regenerative activity, and are resistant to the myotoxicity of local anesthetics (Kalhovde et al., 2005; McLoon et al., 2007). Determining whether these differences in satellite cells are intrinsic and how they contribute to regenerative potential is unclear, however. In one comparison of satellite cells between the masseter and limb, there was no difference with regards to myogenic potential *in vitro* (Grefte et al., 2012). Another study showed that masseteric satellite cells differentiated more slowly, but contributed to limb muscle regeneration *in vivo* (Ono et al., 2010). Limb satellite cells have not been studied in models of facial muscle injury and so the prospects for using limb muscle stem cells to regenerate facial muscles are not yet defined.

Assessment of regenerative potential from satellite cells must include analysis of both the satellite cell proliferative response and the regenerated muscle fiber type and function. Skeletal muscles and craniofacial muscles differ in the myosin isoforms that they express. The muscle fibers of the face express embryonic and neonatal myosin in addition to adult myosin isoforms. Occasionally, facial muscles express multiple myosin isoforms within a single muscle fiber, which has not been observed in other muscles (Stal, 1994; Porter, 2002). Distinct myosin isoforms and a greater number of mitochondria in craniofacial muscle cells may contribute to the resistance to fatigue that craniofacial muscles exhibit. Assuming these unique characteristics of craniofacial muscles are important to their structure or function, recreating these nuances using body muscle stem cells may not be straightforward.

Finally, craniofacial muscles exhibit different susceptibility to pathological conditions. In diseases such as amyotrophic lateral sclerosis (OMIM 105400), the extraocular muscles are not affected. Other craniofacial muscles such as the masseter are affected less severely than body skeletal muscles (Valdez et al., 2012). In contrast, diseases such as myasthenia gravis (OMIM 254200), oculopharyngeal muscular dystrophy (OMIM 164300), and chronic progressive external ophthalmoplegia (OMIM 157640) preferentially affect the extraocular and facial muscles (Benveniste et al., 2005; Greaves et al., 2010).

With regards to regenerative strategies for muscles, the importance of the differences between craniofacial and body muscles in developmental origins, satellite cells, and contractile elements is unclear. The phenotypic differences between extraocular, masseteric, and limb skeletal muscle may be important for regenerating muscle for craniofacial diseases. It is unknown whether satellite cells from the same muscle subset are required to achieve the same phenotype, or whether transplanted satellite cells will adopt the phenotype of their new environment. The answers to these questions could have critical implications for the treatment of muscle-group specific dystrophies. For example, if satellite cells retain adequate intrinsic plasticity, one potential regenerative strategy would be to use autologous transplantation of cells from unaffected or less affected muscle groups to more severely affected muscles. Similarly, satellite cells could be harvested from expendable muscles of the body to regenerate craniofacial muscle defects, with the goal of achieving function in addition to form.

Innervation to the different groups of facial muscles is by the cranial nerves, which have a highly conserved organization among vertebrates. Unlike spinal motor neurons, stemming from columns along the spine, cranial motor neurons extend from discrete nuclei in the midbrain and hindbrain (reviewed in Gilland and Baker, 2005; Guthrie, 2007). Each cranial motor nerve innervates a large number of distinct muscles, many of which can be controlled individually. Some cranial nerves are strictly efferent motor neurons, including cranial nerves III, IV, and VI to the extraocular muscles and cranial nerve XII controlling tongue movement. Other cranial nerves are mixed with motor and sensory components. These “branchiomeric” nerves have sensory ganglia that are formed by contributions from neural crest cells (Figure 1), and motor components that extend to striated muscles as well as to parasympathetic ganglia (cranial nerves III, VII, IX, and X), and the mechanosensory hair cells of the inner ear (cranial nerve VIII; reviewed in Guthrie, 2007). Also unique in craniofacial nervous system development is the development of the sensory organs from the cranial placodes (reviewed in Streit, 2004; Schlosser, 2006). Despite the unique organization and development of the cranial nerves, however, they appear to be functionally similar to other peripheral nerves of the body and there is no known difference in their regenerative capacity.

In the following sections, we present clinical vignettes to illustrate typical craniofacial disorders and how regenerative approaches may be applied in order to treat the conditions.

## Treatment Options for Craniofacial Bone Reconstruction Depend on the Characteristics of the Defect and the Patient’s Age

### Clinical vignettes

**Figure 2:**
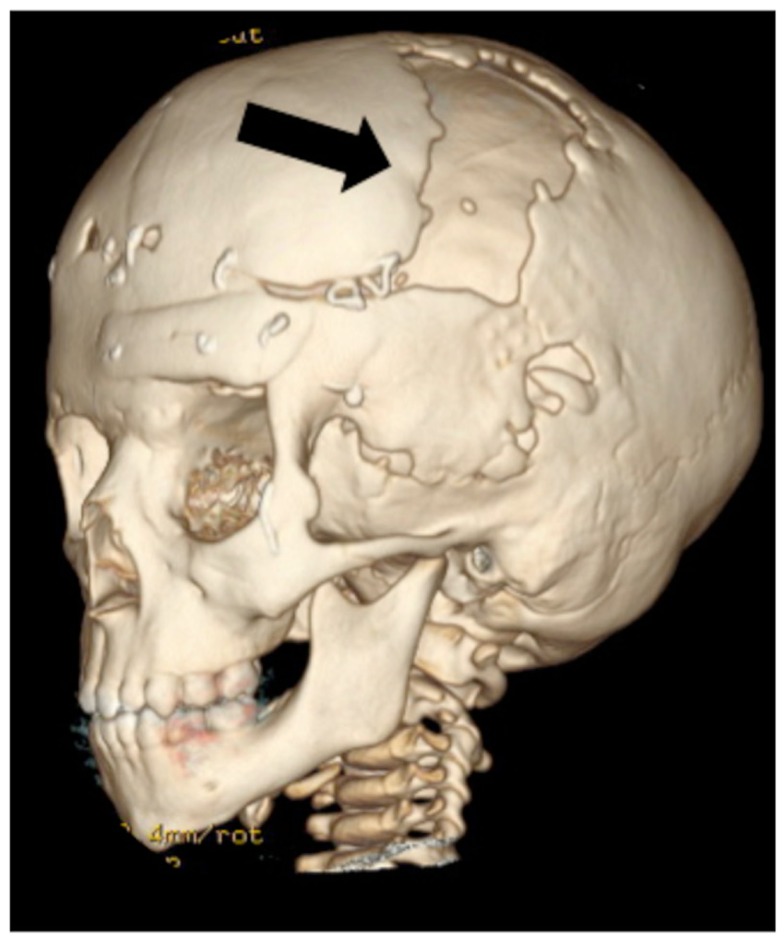
**A computed tomography scan demonstrates a large defect (arrow) in the left frontoparietal skull of a 3-year-old boy**. This required reconstruction with alloplastic materials or large bone grafts.

A boy presented with a large post-operative cranial defect after treatment for coronal suture craniosynostosis (Figure 2). Although defects of this size usually are replaced by regenerated bone in infants, the chance of regenerating this type of defect is low in children older than 2 years. This patient was 3-years-old, and he therefore required reconstruction with prosthetic material.

**Figure 3:**
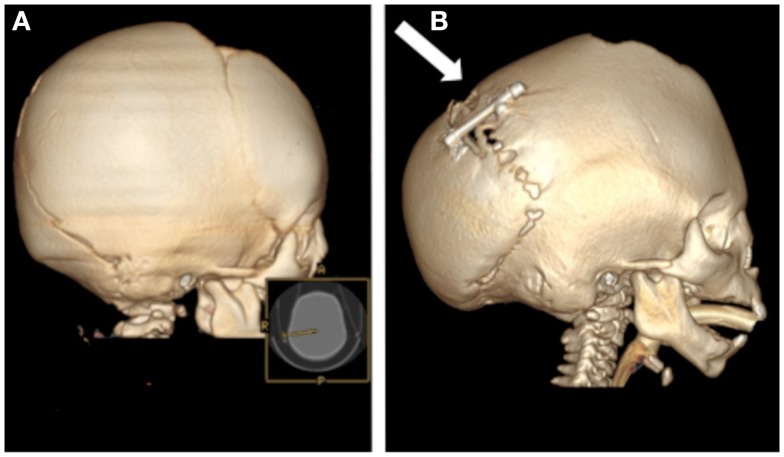
**Computed tomography scans of an infant with multiple suture synostosis preoperatively (A) and 4 months after distraction of the posterior cranial vault (B)**. The distraction footplates have been gradually separated by a distance of 25 mm and evidence of calcified bony regenerate is present between the footplates (arrow).

Another infant with multiple suture synostosis had elevated intracranial pressure due to premature closure of the cranial sutures (Figure 3A). To increase the size of the posterior cranial vault and decrease the intracranial pressure, the child was treated with distraction osteogenesis. After creating osteotomies and placing a distraction device, the occipital bone was gradually advanced posteriorly and new bone gradually regenerated to fill the defect. Regeneration is extensive, but calcification incomplete, after 4 months (Figure 3B).

Craniofacial bones are responsible for bearing the forces associated with mastication, supporting the structures of the face, and protecting the brain and orbits. In adult mammals, bony defects of a critical-size will not regenerate normally and typically require reconstruction. Critical-size bony defects [8 mm in rats (Takagi and Urist, 1982), 15 mm in rabbits (Dodde et al., 2000), and 30 mm in sheep (Reichert et al., 2009)] will not regenerate over the lifetime of the adult animal. Reconstruction of such skull defects typically requires alloplastic materials or bone grafting.

In contrast to adults, infants successfully regenerate bone in large cranial defects. One common example in which this occurs is after surgical treatment for craniosynostosis. In this operation, large bony gaps are created to expand the skull and permit brain growth. Remarkably, when cranial vault expansion is performed before one year of age, normal cranial bone is regenerated to fill the large iatrogenic defects. In the clinical scenario described in Figure 2, the patient required alloplastic reconstruction given his relatively advanced age of 3 years. The mechanisms behind age-related differences in skull regenerative capacity remain poorly understood. Cranial bone regeneration is thought to occur by osteoinduction from the underlying dura (Hobar et al., 1993). Therefore, identifying how dural signaling changes after infancy would presumably shed light on how the regenerative capacity of cranial bone changes with age. Currently available treatments to replace bone meet the functional requirements of cranial bone. However, regenerative strategies could theoretically improve outcomes in certain scenarios by preventing the need to use prosthetic materials or obviating complications such as failed bone grafts, infection, and donor site morbidity. One theoretically attractive avenue would be to restore the mechanisms that allow full regeneration of cranial bone in infants and apply these principles to older patients (Wan et al., 2008).

A highly effective treatment for craniofacial bony defects and deficiencies is distraction osteogenesis, which induces bone generation (reviewed in McCarthy et al., 2001). In this procedure, an osteotomy is made in the area of desired bone generation, and early fracture healing leads to callous formation. The fibrovascular matrix that comprises the callus is then lengthened by gradual mechanical strain in the desired direction of growth. Osteoblasts secrete osteoid to fill the bony gap, and after complete mineralization and bony remodeling, the histology of this new bone resembles that of normal bone (Alman et al., 2011). Strength of the bone after distraction osteogenesis, however, was approximately forty percent less than normal bone when studied in the mandible (Schwarz et al., 2010). This technique, first developed in long bones, has been effectively used to lengthen the mandible in cases of mandibular hypoplasia (Ow and Cheung, 2008), or expand the cranial vault in cases of complex craniosynostosis (Figure 3; Taylor et al., 2012). While distraction osteogenesis was initially developed for linear vectors of growth, strategies are being developed to apply distraction to the complex shapes of the facial skeleton, via use of multi-directional distraction devices (Schendel, 2011).

Distraction and bone grafting offer very effective treatments for craniofacial bony deficiencies. In fact, one may argue whether additional “regenerative” approaches are warranted. The strongest argument in favor of developing new approaches relates to the morbidity of the current solutions, which can be considerable with distraction and procedures that involve harvesting of bone grafts. Another important issue of relevance is the need for approaches that produce bone that will grow with the patient. Such advances would eliminate the need to delay or repeat treatments.

Additional strategies for regenerating bone include the use of growth factors or stem cells. Bone morphogenetic proteins (BMPs) have enhanced effective osteogenesis and improved healing potential in critical-size calvarial defects (Sato and Urist, 1985; Lindholm et al., 1988). BMPs were approved for use in the US in 2004, with approved indications including tibial fractures, sinus augmentations, alveolar ridge augmentations, and lumbar spinal fusions. The complication rate associated with the use of BMPs has recently called into question the use of BMP, however (Williams et al., 2011). This highlights the complexity associated with “targeted molecular” approaches to induce bone formation. Other growth factors such as transforming growth factor-β (TGF-β), and platelet-derived growth factor (PDGF) may also contribute to improved regeneration potential in the appropriate environment (Schilephake, 2002). Given the unique embryologic origins of cranial bone, it is reasonable to assume that the response of cranial osteoblasts to particular growth factors could differ from the response of long bone osteoblasts. Furthermore, the importance of mechanical forces in bone healing may play a large role in the healing potential of bone given the unique processes by which cranial and axial skeletal bone form (i.e., endochondral vs. intramembranous ossification). These unique characteristics of cranial bone compared with the axial skeleton must be taken into account as sophisticated methods of inducing bone regeneration are investigated and developed.

With regards to cell-based approaches, both bone marrow-derived mesenchymal stem cells and adipose-derived mesenchymal stem cells have been demonstrated to form bone *in vitro* (Jaiswal et al., 1997; Zuk et al., 2001; Dragoo et al., 2003; Hicok et al., 2004) and *in vivo* when delivered in conjunction with scaffolds. Some studies demonstrated that the regenerated bone was histologically comparable to surrounding bone (Cowan et al., 2004; reviewed in Zuk, 2008). A variety of precursor cells can be differentiated into osteogenic cells *in vitro* (reviewed in Mao et al., 2006; Seong et al., 2010). Current research also attempts to further understand how different combinations of scaffolds, cells, and growth factors may improve bony regeneration of craniofacial structures. Despite the large body of basic science evidence supporting some of these strategies, few have made their way into common clinical practice. Clinical trials are underway investigating the use of particular bone marrow fractions to induce or enhance alveolar bone regeneration with subsequent dental implants (Kaigler et al., 2012; ClinicalTrials.gov ID NCT01616953). However, because many successful clinical tools are already available, morbidity is acceptable, and outcomes are generally good, these new approaches would need to present much improved function, safety, cost, and decreased morbidity in order to be widely adopted.

## Stable Restoration of Facial Contour by Transplantation of Adipose Tissue

### Clinical vignette

**Figure 4:**
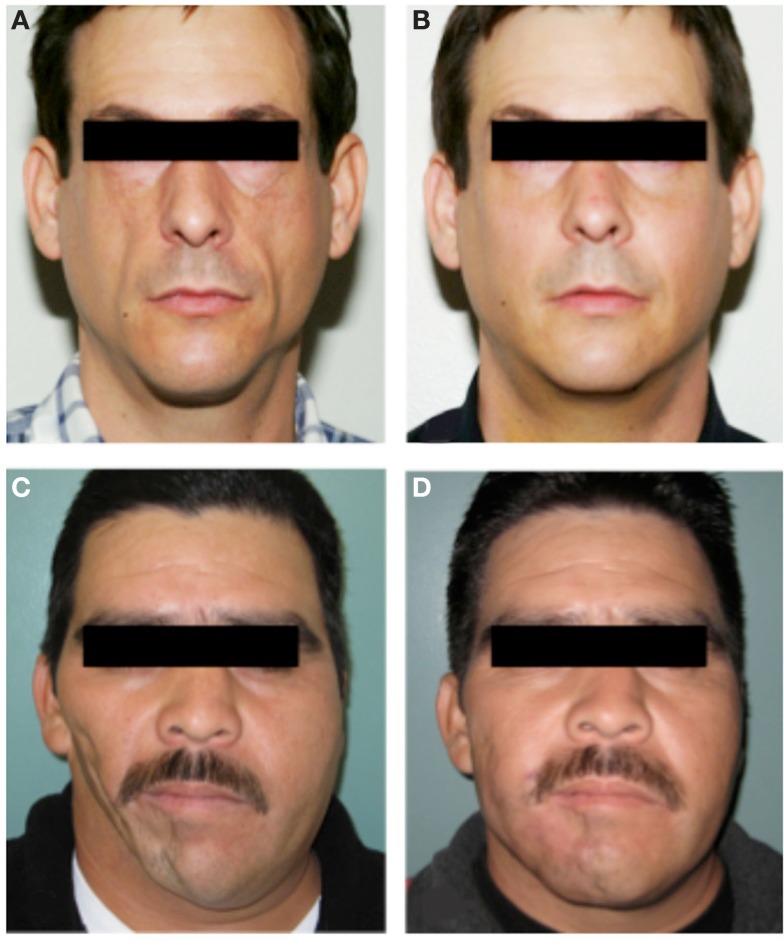
**Autologous fat transfer to treat facial soft tissue deficiency**. A man with severe HIV lipodystrophy [**(A)**, preoperative photo] underwent serial fat grafting to both malar regions [**(B)**, post-operative photo]. This restored normal facial contour and a more youthful appearance. **(C)** Preoperative photo of a patient with post-traumatic soft tissue atrophy on the right side of his face had long lasting improvements in facial symmetry after several sessions of fat grafting from the abdomen to the right cheek and jaw region [**(D)**, post-operative photo). In both cases lipoaspirate was processed by brief centrifugation and passage through a syringe. The cells within the lipoaspirate were not altered or enriched for specific cell types. Multiple injections of very small quantities of fat were used in each treatment.

A man with HIV lipodystrophy presented with severe hollowing in the cheeks due to atrophy of the malar fat pads (Figure 4A). Another man with a remote history of trauma to the right side of his face developed progressive soft tissue atrophy leading to severe facial asymmetry (Figure 4C). Both men underwent several sessions of autologous fat grafting to restore more normal volume and contour to their faces (Figures 4B,D).

Fat grafting for both reconstruction and rejuvenation of the aging face has increased in popularity in the last 10 years, but fat grafting has been used in various forms for many decades. Autologous fat grafting has a number of theoretical and observed advantages that are rapidly making it the preferred approach for facial augmentation. The use of autologous living tissue has the following benefits: incorporation of a living graft into the surrounding tissues, minimal chance of infection, and a natural appearance and feel that is distinctly better than implants and most fillers. Remarkably, grafted fat not only creates volume, but its integration as a living tissue can result in beneficial interactions with surrounding tissues. For example, grafting of fat into an area of contracted, and/or irradiated, skin results in softening, improvements in elasticity, and increased health of the overlying and surrounding skin (Klinger et al., 2008; Mojallal et al., 2009; Phulpin et al., 2009). The mechanism by which grafted fat improves the quality of adjacent skin is unknown, but may involve improved vascularization or secreted paracrine factor effects. In contrast, prosthetic materials or fillers can have undesirable interactions with the surrounding tissue. At best, these materials are relatively inert. However, as a foreign material, fillers are susceptible to causing inflammatory reactions, allergies, or infection (reviewed in Hirsch and Stier, 2008).

Another challenge with soft tissue augmentation relates to the duration of the augmentation effect. Implants can be permanent in the absence of complications. However, implants may also require repositioning or replacement over time and are susceptible to capsular contracture. Synthetic or natural fillers are temporary and typically last only several months. These fillers require repeated treatments and considerable associated financial cost. Long-term studies have now shown that autologous fat grafting can last decades or longer (reviewed in Coleman, 2006a,b), offering another major benefit over the impermanence of synthetic or natural fillers. At present, the most important issue facing the wide adoption of fat grafting, however, is the wide variability in techniques and results among different practitioners. After fat grafting, the retention of fat volume ranges from 20 to 90% in various studies (reviewed in Wetterau et al., 2012). Furthermore, the biology of fat grafting with regards to the stem cell sources of adipocytes, how engraftment occurs, and the factors that influence graft retention are not yet fully understood (Bucky and Percec, 2008).

Adipose tissue contains a robust source of adipose stem cells, and has a high rate of endogenous turnover. Approximately 50% of adipocytes in the body are replaced every 8 years (Spalding et al., 2008), although this has not been studied in craniofacial fat specifically. Preadipocytes are capable of self-renewal and differentiation into white adipose tissue, but are committed to a single cell fate prenatally or in the early postnatal period (Tang et al., 2008). In addition to preadipocytes, the stromovascular fraction of lipoaspirates contains cell populations capable of differentiation into fat, bone, muscle, and cartilage *in vitro* (Zuk, 2008). However, the precise relationship of these cells to committed preadipocytes is not entirely clear (reviewed in Cawthorn et al., 2012). Some clinicians advocate the isolation of these cells in the stromovascular fraction to augment the lipoaspirate in fat grafting (Yoshimura et al., 2009), based on the notion that adipose-derived mesenchymal stem cells in the stromovascular fraction secrete angiogenic growth factors, which may increase graft survival. Other growth factors such as insulin, insulin-like growth factor-1 (IGF-1; Yuksel et al., 2000), and platelet-rich plasma (Nakamura et al., 2010; Pires Fraga et al., 2010) have also been added to fat grafts to improve retention, with greater final fat graft weight, and vascularization as compared with untreated grafts in animal models.

Clinically, currently available fat grafting strategies are very successful for the treatment of contour deformities from lipodystrophies and rejuvenation of the aging face. The observed stability of fat transfer over the long-term strongly suggests that fat regeneration occurs within the graft, with continued differentiation of adipocytes from preadipocytes and normal fat turnover. This normal tissue homeostasis involving the continuous generation of new fat cells is evidence of the existence of a tissue-resident stem cell for fat. It follows that current fat transfer techniques are, in fact, transferring adipose stem cells along with adipocytes and other cell types. Fat grafting, therefore, largely fits the definition of regenerative medicine. Augmenting a fat graft with particular purified cell fractions or growth factors may hold promise for improving predictability and retention, although clear superiority of these techniques compared with traditional methods has not been shown. Clinical trials are underway to more critically evaluate whether concentrating the stromovascular fraction in lipoaspirates will be better than traditional methods in treating post-traumatic soft tissue deformities of the face (ClinicalTrials.gov ID NCT01564524).

The cellular mechanisms contributing to lipodystrophies and aging are not fully understood, however overcoming the gaps in knowledge about fat biology and pathology would potentially allow direct regeneration of fat without grafting from other sites. Currently adipose precursor cells have been shown to form fat *in vitro* (Kim et al., 2007; Wu et al., 2012), but direct fat differentiation *in vivo* has not been reported. A clinical trial to assess efficacy of adipose-derived stem cell injections in progressive hemifacial atrophy (ClinicalTrials.gov ID NCT01309061) is in progress. At present, however, the indications for fat grafting continue to expand and clinical results continue to improve.

## Permanent Diplopia after Injury to an Extraocular Muscle

### Clinical vignette

**Figure 5:**
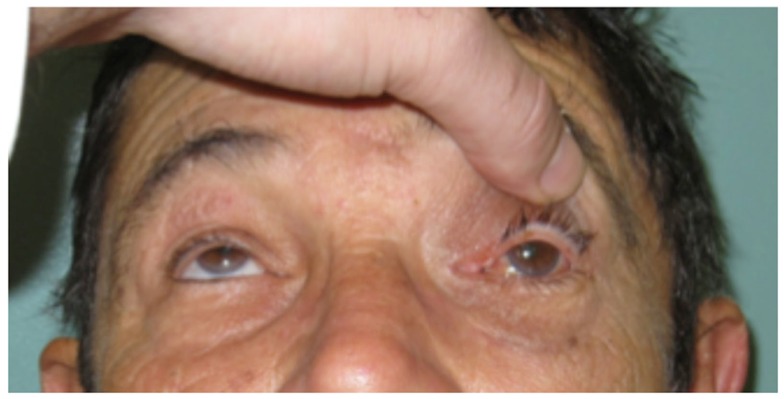
**This man suffers from the inability to look upward with his left eye after permanent injury to the left superior rectus muscle**.

An elderly man suffered an orbital roof fracture that caused entrapment of his left superior rectus muscle. This injury left him with diplopia (double vision) and an inability to look upward with his left eye (Figure 5). Despite release of the muscle from the fracture fragments, the injury to the muscle was permanent due to muscle fibrosis.

While reasonably good techniques exist for replacing bone and fat, regenerating, repairing, or replacing functional muscle remains a significant challenge. Although muscle transfers (called flaps) have vastly improved our ability to treat a variety of tissue defects over the past three to four decades, there remain major limitations in the function that can be achieved. Strategies for addressing the loss of fine muscle function, including the critical functions of many small muscles of the face and head, are lacking. Myoblasts, or muscle precursor cells, have been injected into injured muscle in animal models and in patients with Duchenne muscular dystrophy to promote muscle regeneration (Rando and Blau, 1994; Miller et al., 1997). However, myoblasts exhibit relatively poor survival and engraftment into the host tissue, and studies in mice now clearly demonstrate the superiority of muscle stem cells (satellite cells) in terms of their ability to engraft and regenerate muscle. Satellite cells in the muscle, bone marrow-derived mesenchymal stem cells, and adipose-derived mesenchymal stem cells have all been shown have myogenic properties *in vitro* (Wakitani et al., 1995; Zuk et al., 2001; Muguruma et al., 2003; Di Rocco et al., 2006). However, mesenchymal stem cells have not demonstrated successful regeneration *in vivo* (reviewed in Otto et al., 2009). Only satellite cells have truly fulfilled the criteria of a stem cell for muscle.

Therefore, satellite cells currently show the most promise in translational applications for functional muscle regeneration. These adult muscle stem cells are capable of robust self-renewal, differentiation into myoblasts, and formation of mature skeletal muscle fibers in response to injury (Bischoff, 1986; Zammit et al., 2006; Cosgrove et al., 2009). Transplantation of intact single myofibers into injured muscle leads to satellite cell renewal and myofiber regeneration (Collins et al., 2005; Hall et al., 2010). Single, prospectively isolated muscle stem cells have been transplanted into mouse muscle, demonstrating self-renewal, expansion, and differentiation into functional muscle fibers *in vivo* (Cerletti et al., 2008; Sacco et al., 2008). Muscle stem cell transplantation has resulted in correction of dystrophic phenotypes in mdx mice (Sacco et al., 2010). Major remaining challenges include the translation of mouse satellite cell biology to humans, and overcoming additional hurdles such as correction of genetic defects and *ex vivo* satellite cell expansion.

As demonstrated by the example of extraocular muscle injury (Figure 5), regeneration of craniofacial muscle is an area of great clinical need. Ideal treatments might involve transplantation of autologous satellite cells from an area of excess to an area of need. Before it becomes a clinical reality, we must better understand the differences between satellite cell populations. Are they capable of regenerating only their native muscle phenotype? Or is it possible that limb satellite cells could effectively regenerate extraocular muscle?

## Reanimating the Face: Regenerative Strategies for Nerve and Muscle in Facial Paralysis

### Clinical vignette

**Figure 6:**
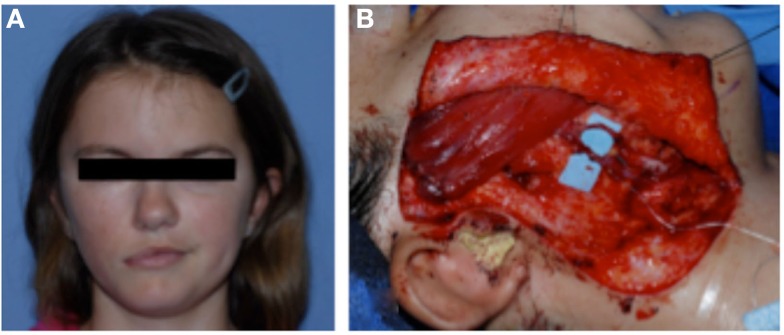
**(A)** A girl with congenital right-sided facial paralysis demonstrates asymmetry with smiling. She was treated with free gracilis muscle transfer. **(B)** The muscle is inset to the zygoma and the oral commissure. The new vascular supply to the muscle is shown on the blue background. The muscle was innervated by the nerve to masseter (not shown).

A girl with congenital right-sided facial paralysis was treated with an innervated muscle flap to restore a functional smile (Figure 6). This procedure involved free gracilis muscle micro-neurovascular transfer to the face. The muscle was innervated by the ipsilateral nerve to the masseter. With clenching of the teeth, the gracilis muscle would contract and elevate the oral commissure to recreate a natural symmetric smile.

Injury to the facial nerve leads to two tissue problems. First, injury to the nerve leads to denervation of the target muscles. Second, denervation of the muscles over the long-term leads to muscular atrophy and loss of function (Kobayashi et al., 1997). After injury, axons degenerate in response to denervation (Sunderland and Bradley, 1950). Peripheral nerves are unable to regenerate the cell body, but axons are able to regrow from existing cells at a rate of 1 mm/day in humans. For this reason, in cases of nerve transection or other injury that will not recover on its own, current treatments aim to restore continuity of the nerve sheath and guide axonal regeneration, either by primary nerve repair or nerve grafting (reviewed in Siemionow et al., 2010). Successful reinnervation can occur, but is dependent upon the location of the injury and the timing of the repair. Improving axon growth in both acute and chronic nerve injury is critical to improving the functional potential of regenerating peripheral nerves.

Schwann cells play an important role in supporting axon growth. They closely accompany the axons as they grow. Schwann cells migrate distally from the zone of injury ahead of the regenerating axon, as if clearing a path for the axon. Schwann cells also secrete laminin, fibronectin, and other factors that facilitate axonal growth (reviewed in Zochodne, 2012). However, denervation of the nerve stump leads to loss of Schwann cells (Sulaiman et al., 2002), and limits regeneration. Therefore, one approach to supporting peripheral nerve regeneration is to transplant Schwann cells. Schwann cells have been expanded in culture and transplanted to chronically denervated rat tibial nerves. These cultured Schwann cells increased axonal regeneration and muscle reinnervation (Walsh et al., 2010). Similarly, adipose-derived stem cells have been differentiated into a Schwann cell phenotype for this purpose (Kingham et al., 2007), and demonstrated myelination and improved nerve regeneration after transplantation distal to a sciatic nerve injury in a rat (Tomita et al., 2012). While these approaches need to be further refined and verified with regards to functional outcomes, Schwann cell transplants may prove successful for peripheral nerve regeneration (reviewed in Walsh and Midha, 2009).

Several challenges must be overcome in peripheral nerve axonal regeneration. First, the rate of growth remains very slow. Finding ways to accelerate the axonal growth rate would decrease the amount of degeneration that both the peripheral nerve axon and the target muscle experience. It is known that advanced age can slow both axonal regeneration (reviewed in Verdu et al., 2000) and collateral sprouting (Kovacic et al., 2010), however there are presently no known mechanisms for accelerating axonal growth. Second, there are innate inhibitory interactions that occur at the regenerating axon (reviewed in Zochodne, 2012). Studying how to overcome these inhibitory pathways to promote axonal growth will also be important in optimizing peripheral nerve regeneration. Second, an additional major challenge is to accurately control the direction of axonal growth. This is a critical problem, noted in particular after inflammatory injury to the facial nerve, as occurs in Bell’s palsy. In patients with Bell’s palsy, synkinesis, or abnormal simultaneous muscle movement, can occur due to aberrant regeneration of the nerve axons. Using either physical or molecular guides to ensure an axon reaches its appropriate target would have tremendous clinical implications.

In addition to the problems associated with axonal regrowth are the subsequent deficits caused by target muscle atrophy. After denervation, muscle mass and contractile force rapidly decrease. The rapid loss stabilizes at approximately 4 months, with the muscle retaining only 25% of its mass and less than 0.1% of its maximum contractile force (reviewed in Carlson, 2004). In the early period after denervation, satellite cells are activated, proliferate, and form new muscle. However, these fibers are morphologically abnormal, small in size, and do not have satellite cells associated with them (Borisov et al., 2005). Finally, after prolonged denervation, the overall number of satellite cells present in the muscle decreases, and the capillary bed degenerates (Borisov et al., 2000; Jejurikar et al., 2002). This phenotype is not surprising given the known dependence of developing muscle on neural input for proper formation and gene expression (Betz et al., 1980; Harris et al., 1989; Crews and Wigston, 1990; Fredette and Landmesser, 1991; Fernandes and Keshishian, 1998). Moreover, limb regeneration in amphibians, including regeneration of the limb muscles, requires innervation (Brockes, 1984, 1987). Therefore, neuromuscular intercellular communication is a prime example of the complex interplay of different tissues, requiring precise orchestration for proper formation during development and in regeneration.

The changes occurring in muscles after denervation further limit the restoration of function, even after axonal regeneration occurs. Preventing the maladaptive changes associated with denervation and reinnervating target muscles more quickly are the major challenges confronting regenerative peripheral nerve biology. For patients with both acute and chronic facial nerve injuries, developing these regenerative strategies may allow for more natural facial function than our current reconstructive treatments are capable of providing.

## Regeneration of Skin after Burn Injury

### Clinical vignette

**Figure 7:**
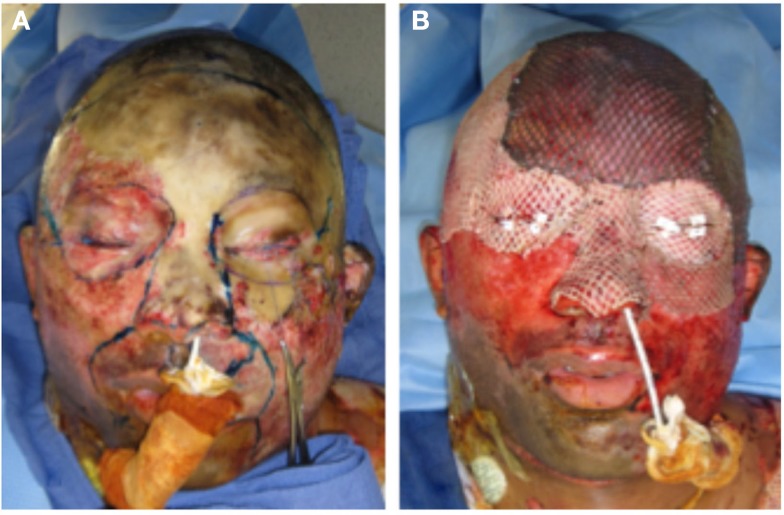
**A young man with full thickness burns of the face and scalp (A) prior to debridement and (B) after cadaveric skin graft placement**. He required multiple operations prior to final skin grafting.

A 22-year-old man involved in an automobile accident suffered full thickness burns to nearly the entire face, scalp, and both upper extremities. His treatment required multiple operations, first for debridement and cadaveric skin grafting to prepare a suitable wound bed prior to autologous grafting (Figure 7). He subsequently had full and split-thickness skin grafts to the face. The scalp had exposed bone and required grafting in two stages: first with artificial dermis (Integra, Integra Life Sciences), then with autologous skin.

An ideal skin replacement in the face would be thin, pliable, similar in color, and texture to surrounding skin, have rapid and reliable engraftment, contain all the components of the skin organ, and undergo minimal contracture and scarring. Autologous skin is the best option currently available, and can be used in several forms. Local skin flaps may cover relatively small defects and provide a good match of skin color and texture. They also do not contract significantly. For larger defects, tissue expansion is successful for increasing the amount of skin available for local rearrangement, but it typically requires two operations separated by several months to recruit adequate skin. It also requires an adjacent donor site with healthy unscarred skin. In more complex cases with both skin and soft tissue deficiency, free tissue transfer of skin with its underlying muscle or fascia will provide excellent coverage. In craniofacial reconstruction, skin flaps often come from a remote location, and reconstruction suffers from poor color and texture match. Finally, in cases such as the burn patient (Figure 7), full or split-thickness skin grafting is the most commonly used strategy for achieving massive amounts of skin coverage. Skin may be harvested from local or remote donor sites and is versatile with regards to its use on both large and small defects. The limitations of autologous skin grafting include donor site availability, donor site morbidity, graft loss, lack of certain dermal elements (sweat glands and hair follicles), and scar contracture. Large burns require multiple operations and serial grafts to finally achieve wound closure, and patients are usually left with significant deformities.

Partial thickness burns retain the components of the skin organ that are responsible for regeneration. The skin has a robust source of stem cells located in the basal layer of the epidermis, the bulge of the hair follicles, and the base of sebaceous glands (Fuchs and Nowak, 2008). Each of these stem cell compartments is capable of forming new epidermis. Alternatives to autologous skin grafts using cells derived from skin stem cells are cultured autologous epidermis and autologous cell suspensions. Cultured autologous epidermis has the advantage that small biopsies may be expanded for large amounts of graftable epidermis. However, these grafts contain an abnormally layered epidermis and, most importantly, lack a dermis. Without a dermis, graft take decreases and scar formation increases, making cultured autologous skin grafts inferior to autologous skin grafts. This is because these grafts lack the elastic properties of a dermal component, resulting in a much more fragile construct, prone to sloughing (Pham et al., 2007). Autologous cell suspensions have potential to improve outcomes with regards to skin quality, color, and rate of healing when used in partial thickness burns (Wood et al., 2012). However, autologous cell suspensions cannot be used to treat more complex full thickness burns for the same reasons detailed above for altered epidermis.

Currently, the most significant hurdle for skin regeneration is the regeneration of the dermis in full thickness and deep partial thickness burns. The dermis is home to the stem cells residing in the hair follicles and sebaceous glands. Loss of the dermis results in an inability to regenerate. Furthermore, the dermis is responsible for the stability of the graft and native skin, elasticity of the skin and prevention of contracture, and important cell-extracellular matrix interactions that are necessary for healing and homeostasis. Without dermis, it is not possible to obtain a stable skin construct that will resist contracture, trauma, and infection.

To address this problem, multiple approaches have been used to engineer artificial dermal matrices. Thus far, collagen-based matrices appear to have better cellular integration than synthetic polymers (reviewed in Widgerow, 2012). Artificial dermis is successful in improving contour and graft take onto bone, cartilage, or tendon (reviewed in Yannas et al., 2011). However, artificial dermis requires the use of autologous skin grafting with some native dermis present in the graft, and does not appear to improve long-term contracture or healing (Philandrianos et al., 2012). A dermal matrix that also contains keratinocytes or basal stem cells and is capable of resurfacing large wounds in one step has yet to be developed. Developing mechanisms for regenerating dermis, or engineering and culturing full thickness skin for grafting, will dramatically change acute burn care. In patients with large areas of full thickness burn in sensitive areas of the face, regenerated skin and dermis could provide greatly improved functional and cosmetic outcomes and allow for treatment to be completed with fewer surgical interventions. Like other tissues, skin is complex, is comprised of multiple cell types, is vascularized by blood vessels, and is innervated. Skin injuries have a great capacity to heal, but the drawbacks of healing are most evident in injuries to the skin of the face. Scarring, deformities, and loss of function are the norm and approaches to “replace like with like” are needed.

In addition to the acute need for skin coverage to prevent infection and fluid loss, burn patients suffer from distinct long-term deformities, such as scar contracture that require additional operations for release. Fat grafting is one strategy that has also been used to soften and improve the quality of scars in burn patients. The molecular mechanisms are unclear, but fat grafting increases the vascularity of the scar and alters its collagen content (Klinger et al., 2008).

## The Future of Regenerative Strategies in Craniofacial Diseases

Two general strategies are emerging as future solutions to craniofacial reconstructive challenges: regenerative approaches discussed in this manuscript and composite tissue transplantation. Composite tissue transplantation has been making inroads in recent years, with the first successful face transplant in 2005 (Devauchelle et al., 2006). At least 18 have been performed worldwide since, including several full face transplants (Pomahac et al., 2012). Facial allotransplantation holds great promise with regards to the restoration of form and function superior to that of traditional reconstructive techniques. One clear advantage of facial allotransplantation is that the complex tissues of the face are fully and normally formed prior to transplantation. Current disadvantages include the need for life-long immunosuppression, with the risks of developing life-threatening infections, and unclear functional integration and cosmetic appearance of the graft. Some facial muscle function and sensation has been documented with facial allotransplantation, but normalization of sensation, expression, and function has yet to be demonstrated with long-term follow up.

Regenerative medicine approaches to regenerate individual functional tissues based on developmental mechanisms may ultimately lead to clinical composite tissue regeneration. A theoretical advantage of this approach is the achievement of fully integrated, complex, functional tissue that is truly “self”-derived. Many unanswered questions exist at this point, including whether function, appearance, and sensation may be better achieved through regeneration of native structures rather than reinnervation of a transplant. The regeneration of complex facial structures also requires precision and specificity. Directing the regeneration of cells such that they proliferate in the appropriate locations at appropriate times, and reach terminal differentiation when the organ is fully regenerated will be challenging. Regeneration strategies will need to develop hand in hand with tissue engineering strategies that allow us to build the components of the face precisely.

## Conflict of Interest Statement

The authors declare that the research was conducted in the absence of any commercial or financial relationships that could be construed as a potential conflict of interest.
